# Thyroid and endostyle development in cyclostomes provides new insights into the evolutionary history of vertebrates

**DOI:** 10.1186/s12915-022-01282-7

**Published:** 2022-04-01

**Authors:** Wataru Takagi, Fumiaki Sugahara, Shinnosuke Higuchi, Rie Kusakabe, Juan Pascual-Anaya, Iori Sato, Yasuhiro Oisi, Nobuhiro Ogawa, Hiroshi Miyanishi, Noritaka Adachi, Susumu Hyodo, Shigeru Kuratani

**Affiliations:** 1 Laboratory of Physiology, Atmosphere and Ocean Research Institute, The University of Tokyo, Kashiwa, 277-8564 Japan; 2 Evolutionary Morphology Laboratory, RIKEN Cluster for Pioneering Research (CPR), Kobe, 650-0047 Japan; 3 Division of Biology, Hyogo College of Medicine, Nishinomiya, 663-8501 Japan; 4 Department of Molecular Biology and Biochemistry, Graduate School of Biomedical & Health Sciences, Hiroshima University, Hiroshima, 734-8553 Japan; 5 Laboratory for Evolutionary Morphology, RIKEN Center for Biosystems Dynamics Research (BDR), Kobe, 650-0047 Japan; 6 Present Address: Department of Animal Biology, Faculty of Science, University of Málaga, Málaga, Spain; 7 Present Address: Andalusian Centre for Nanomedicine and Biotechnology (BIONAND), Málaga, Spain; 8 Laboratory for Haptic Perception and Cognitive Physiology, RIKEN Center for Brain Science, Wako, 351-0198 Japan; 9 Laboratory Research Support Section, Center for Cooperative Research Promotion, Atmosphere and Ocean Research Institute, The University of Tokyo, Kashiwa, 277-8564 Japan; 10 Faculty of Agriculture, University of Miyazaki, Gakuen-kibanadai-nishi, 889-2192 Japan; 11 Aix-Marseille Université, IBDM, CNRS UMR 7288, Marseille, France; 12 Present address: Department of Molecular Craniofacial Embryology, Graduate School of Medical and Dental Sciences, Tokyo Medical and Dental University (TMDU), Tokyo, 113-8549 Japan

**Keywords:** Endostyle, Thyroid gland, Cyclostomes, Hagfish, Lamprey, Evolution, Development, Atavism

## Abstract

**Background:**

The endostyle is an epithelial exocrine gland found in non-vertebrate chordates (amphioxi and tunicates) and the larvae of modern lampreys. It is generally considered to be an evolutionary precursor of the thyroid gland of vertebrates. Transformation of the endostyle into the thyroid gland during the metamorphosis of lampreys is thus deemed to be a recapitulation of a past event in vertebrate evolution. In 1906, Stockard reported that the thyroid gland in hagfish, the sister cyclostome group of lampreys, develops through an endostyle-like primordium, strongly supporting the plesiomorphy of the lamprey endostyle. However, the findings in hagfish thyroid development were solely based on this single study, and these have not been confirmed by modern molecular, genetic, and morphological data pertaining to hagfish thyroid development over the last century.

**Results:**

Here, we showed that the thyroid gland of hagfish undergoes direct development from the ventrorostral pharyngeal endoderm, where the previously described endostyle-like primordium was not found. The developmental pattern of the hagfish thyroid, including histological features and regulatory gene expression profiles, closely resembles that found in modern jawed vertebrates (gnathostomes). Meanwhile, as opposed to gnathostomes but similar to non-vertebrate chordates, lamprey and hagfish share a broad expression domain of *Nkx2-1/2-4*, a key regulatory gene, in the pharyngeal epithelium during early developmental stages.

**Conclusions:**

Based on the direct development of the thyroid gland both in hagfish and gnathostomes, and the shared expression profile of thyroid-related transcription factors in the cyclostomes, we challenge the plesiomorphic status of the lamprey endostyle and propose an alternative hypothesis where the lamprey endostyle could be obtained secondarily in crown lampreys.

**Supplementary Information:**

The online version contains supplementary material available at 10.1186/s12915-022-01282-7.

## Background

The endostyle or thyroid is a defining characteristic of chordates [1–3] (Additional file 1: Fig. S1) [4–7]. The thyroid gland is a major endocrine organ that produces and secretes thyroid hormones (i.e., triiodothyronine and thyroxine) in all vertebrates. The thyroid hormones exert important pleiotropic effects on development, growth, energy metabolism, and metamorphosis. Although the general anatomy of the vertebrate thyroid gland is highly diverse (Additional file 1: Fig. S1A), the thyroid follicle is a common functional unit and an evolutionary novelty of vertebrates [3]. Acquisition of the follicular thyroid gland enables the efficient uptake and storage of iodine, which is notoriously scarce in freshwater environments, likely contributing to the early radiation of aquatic vertebrates [8].

**Fig. 1:**
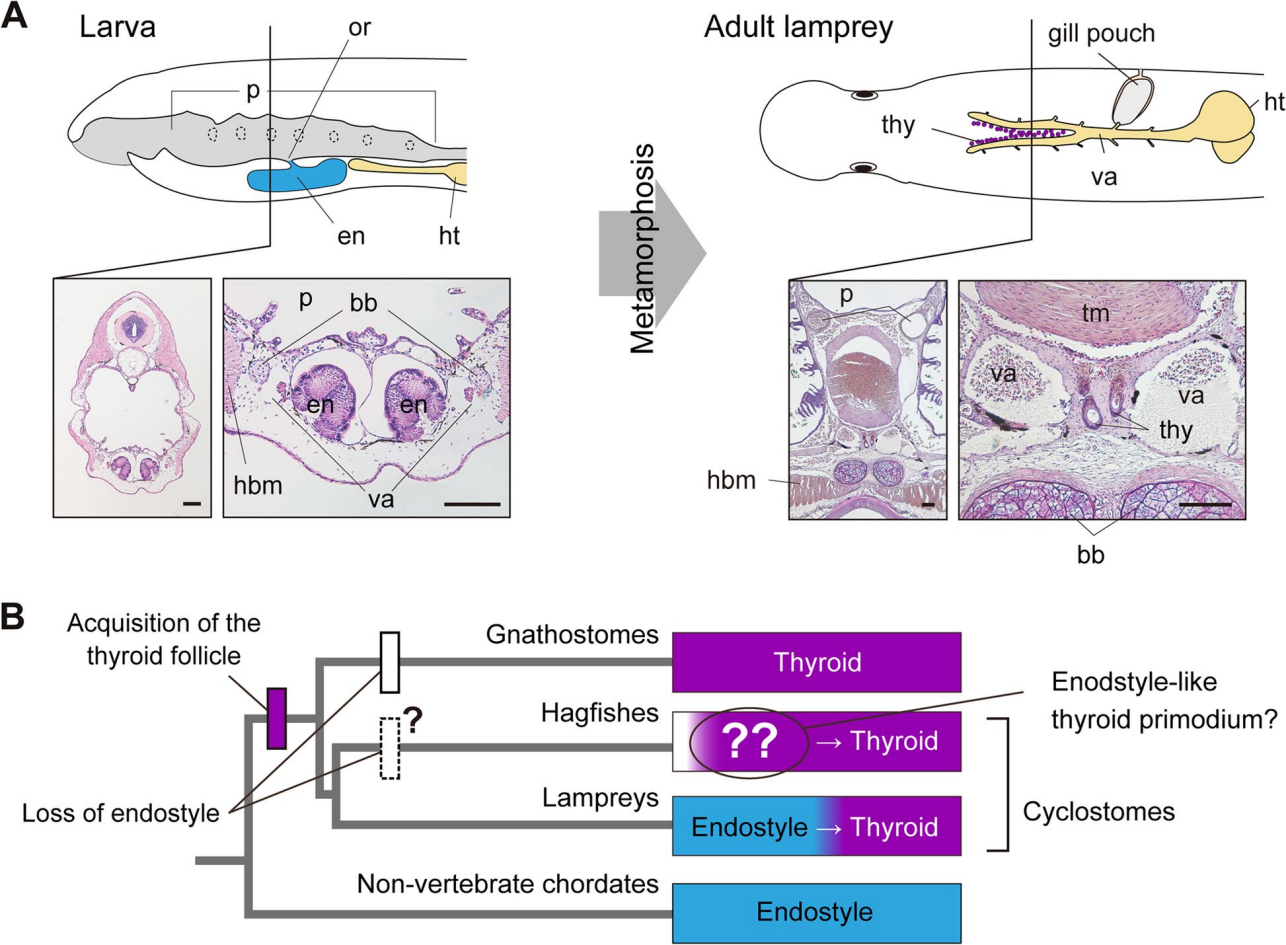
Transition of the endostyle to the thyroid in lampreys and classical evolutionary hypothesis of the vertebrate thyroid gland. **A** Larval lampreys (lateral view) possess an exocrine endostyle (en), which transforms into thyroid follicles (thy) in the adult (ventral view). p, pharynx; ht, heart; or, orifice; bb, branchial basket; va, ventral aorta; hbm, hypobranchial muscle; tm, tongue muscle. Bars, 100 μm. **B** Classical evolutionary hypothesis of the relationship between the endostyle and the thyroid based on the previous studies. Although an endostyle-like thyroid primordium was observed in developing hagfish (*Eptatretus stoutii*) embryos in 1906, it has not been verified ever since [15]. The primitive endostyle has been retained only in modern lampreys but was independently lost in the gnathostome and possibly in hagfish.

The endostyle is the postulated evolutionary precursor of the vertebrate thyroid, occurring in filter-feeding non-vertebrate chordates in an anatomical position similar to that of the thyroid [8–10] (Additional file 1: Fig. S1B). The evolutionary link between the thyroid and the endostyle is supported by many shared morphological, physiological, and developmental traits including accumulation of iodide and peroxidase activity [8, 11], endodermal origin [9], and gene expression profiles [12, 13]. All extant non-vertebrate chordates are microphagous suspension feeders that secrete mucus from the endostyle to capture small particles, such as phytoplankton or detritus. The endostyle is also found in the filter-feeding ammocoete larvae of modern lampreys, and a portion of the epithelial cells of the ammocoete endostyle re-differentiates and transforms into thyroid follicles during metamorphosis [14] (Fig. 1A). Thus, the lamprey endostyle was considered to represent a state of non-vertebrate chordates [1, 8].

Considering the monophyly of cyclostomes [16, 17], further studies on the development of the thyroid in hagfish, the other group of extant cyclostomes, may enhance our knowledge regarding the evolution of the vertebrate thyroid and endostyle by providing a clearer understanding of the last common ancestor (LCA) of vertebrates. In contrast to the many developmental studies in lampreys, the only study on hagfish thyroid development was conducted by Stockard [15], who used *Eptatretus stoutii* (formerly *Bdellostoma stouti*) embryos. He described that the thyroid first appeared as a longitudinal “trough” in the pharyngeal floor [15] (Additional file 1: Fig. S2A-D). The presence of this structure, which was described as a rudiment of the endostyle almost a century later [10], and the broad pharyngeal origin of hagfish thyroid follicles would be consistent with the classical assumption that considers the endostyle a plesiomorphic trait retained in cyclostomes (Fig. 1B). However, mainly owing to the difficulty in obtaining and maintaining fertilized hagfish eggs [18], no molecular, genetic, or morphological data pertaining to hagfish thyroid development have been updated over the last century.

In gnathostomes, the shared developmental origin of the thyroid is an endodermal thickening at the level of the first pharyngeal pouch close to the endothelial lining of the anterior end of the fused endocardial tubes (aortic sac) [3, 19]. Loss-of-function studies in mice and zebrafish have revealed several transcription factors that are crucial for thyroid development. Among them, *Nkx2-1* (previously termed *thyroid transcription factor-1*), *Pax8*, and *Hhex* form a mutually dependent gene regulatory network (GRN) [19, 20]. Although these transcription factors are not required for thyroid specification, the deletion of any of these resulted in athyreosis or severe thyroid hypoplasia, suggesting that the formation of the GRN is essential for the thyroid organogenesis [19, 21]. *Foxe1* (previously termed *thyroid transcription factor-2*), downstream of the GRN, is also known to be essential for thyroid development in mammals, whereas the thyroid develops normally (with no changes in its morphology) in knockdown experiments in zebrafish embryos injected with a *Foxe1* morpholino [22]. This suggests that the regulatory roles of *Foxe1* in thyroid development may not be conserved among gnathostomes [19].

Here, we observed staged embryos of the Japanese inshore hagfish (*Eptatretus burgeri*), provided an updated description of thyroid development in detail, and deduced the direct development of the thyroid as in gnathostomes. Furthermore, to compare molecular developmental signatures between the development of thyroids in hagfish and gnathostome and that of the endostyle in lamprey, we examined expression profiles of orthologs of the abovementioned thyroid transcription factor genes (*Nkx2-1*, *Pax8*, and *Hhex*) during the development of *E. burgeri*, the cloudy catshark (*Scyliorhinus torazame*), and the Arctic lamprey (*Lethenteron camtschaticum*).

## Results and discussion

**Fig. 2:**
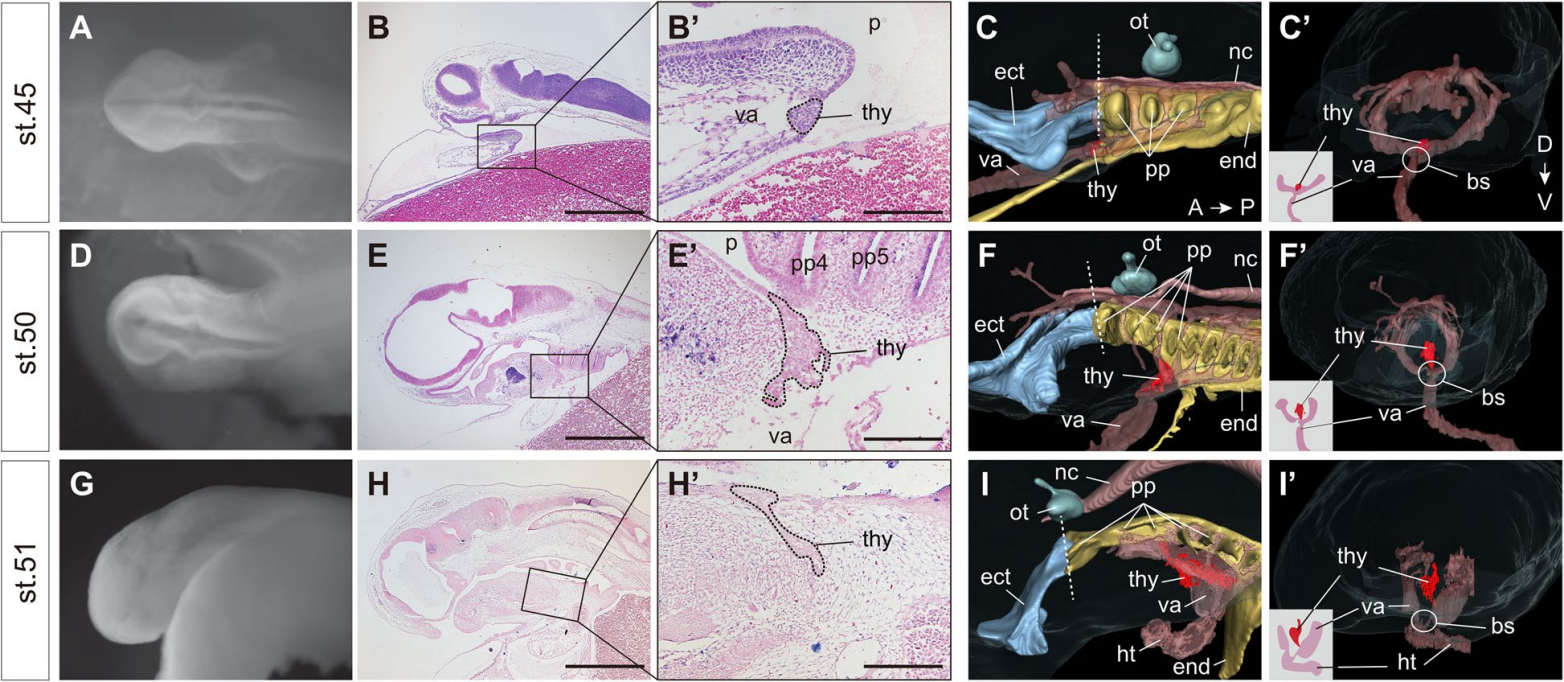
Development of the thyroid gland in the *Eptatretus burgeri* embryo. Dorsal (**A**, **D**) and lateral views (**G**) of fixed embryos immersed in methyl benzoate prior to paraffin embedding. **B**, **B’**, **E**, **E’**, **H**, **H’** Midline sagittal sections of the hagfish thyroid. **B**, **B’** Sections were stained using hematoxylin and eosin. **E**, **E’**, **H**, **H’** Sections including thyroid primordia were stained via in situ hybridization of *Tbx1/10A* (**E**, **E’**) and *Lhx3/4A* (**H**, **H’**) with nuclear fast red in a previous study [17]. The area surrounded by dashed lines indicates thyroid primordium. Lateral (**C**, **F**, **I**) and frontal (**C’**, **F’**, **I’**) views of 3D reconstructed images. The ectoderm and endoderm are colored light blue and yellow, respectively. Dashed lines indicate the boundary between stomodeum and pharynx. Thyroid is colored red. Both cardiac field and blood vessel are transparently colored light red. pp, pharyngeal pouch; ect, ectoderm; nc, notochord; ot, otic vesicle; end, endoderm; heart, ht; ventral aorta, va; bs, bifurcation site of ventral aorta. See Fig. 1 for other abbreviations. Bars, 1 mm (**B**, **E**, **H**) and 200 μm (**B’**, **E’**, **H’**)

Contrary to previous observations of *E. stoutii*, we did not observe a trough-like structure during any of the developmental stages of *E. burgeri* (Fig. 2 and Additional file 1: Fig. S2E-G). Instead, we found a putative thyroid anlage in the form of a thickened epithelium on the pharyngeal floor juxtaposed to the aortic sac during the early pharyngula stage [17, 23] (at stage 45; Fig. 2A–C’), which is considerably earlier than the stage wherein the trough-like structure was observed by Stockard (at stage 53) [15]. Since the ventral pharynx of the hagfish embryo is rostroventrally everted in early development, the position of the hagfish thyroid anlage is difficult to identify based on the level of the pharyngeal arches [10]. Nevertheless, this thyroid anlage arose from a focal region of the pharyngeal floor slightly posterior to the root of the lower lip anlage and did not extend throughout the entire gill region as previously reported [15] (Fig. 2B, B’, C). The collection of hagfish embryos prepared by Bashford Dean, which was subsequently used in Stockard’s histological studies, showed a frequent distortion of embryonic tissues [24, 25]. The trough-like organ described by Stockard was not a consistent observation; thus, it was likely an artifact due to flawed fixation.

**Fig. 3:**
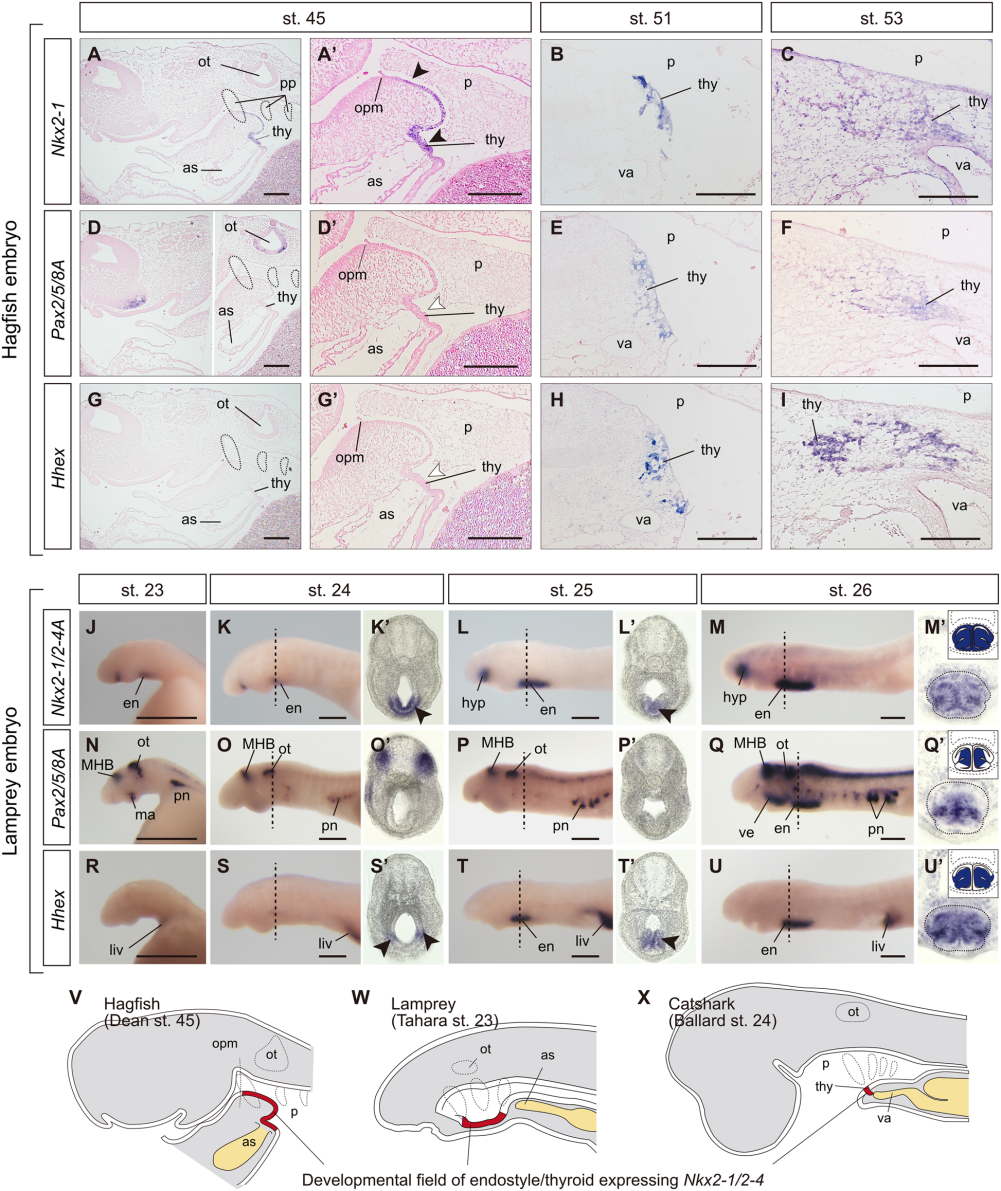
Expression profiles of the genes involved in thyroid development in modern cyclostomes. **A**–**I** and **J**–**U’** hagfish and lamprey embryos, respectively. **V**, **W**, **X** schematic of the *Nkx2-1/2-4* expressing domain (colored red) in hagfish, lamprey, and catshark embryos when the expression of *Nkx2-1/2-4* first detected in this study (stages 45, 23, and 24, respectively). **A’**, **D’**, **G’**, **K’**, **L’**, **S’**, **T’** Filled and open arrowheads indicate the presence and absence of signals, respectively. **K’**, **L’**, **M’**, **O’**, **P’**, **Q’**, **S’**, **T**’, and **U’** Transverse sections of hybridized embryos in the plane of the dashed lines. **M’**, **Q’**, **U’** Diagram of gene expression in the endostyle is shown in the boxes. as, aortic sac; opm, vestigial oropharyngeal membrane; MHB, midbrain-hindbrain boundary; ma, mandibular arch; liv, liver; hyp, hypothalamus; ve, velum; pn, pronephros. See Figs. 1 and 2 for other abbreviations. Bars, 200 μm

The position of the thyroid primordium in the hagfish embryo from stages 45 to 51 was continuously associated with a bifurcation site of the ventral aorta probably including the precardiac mesoderm (Fig. 2C’, F’, I’). In gnathostomes, the thyroid develops from an endodermal thickening of the pharyngeal floor in close vicinity to the aortic sac [3, 19], as was observed in the cloudy catshark (*Scyliorhinus torazame*; Fig. 3X; Additional file 1: Fig. S3A’-E’). Furthermore, in mice, frogs, and zebrafish, extrinsic factors, such as FGF2 and BMP4, presumably secreted from the precardiac mesoderm, have been shown to induce thyroid fate [19, 26]. Unlike the initiation of the thyroid development in hagfish and gnathostomes, evagination of the lamprey endostyle primordium from the midline pharyngeal endoderm occurs from the 2nd to 4th pharyngeal arches, and only the posterior end of the endostyle is in contact with the bifurcation site of the aortic sac [2] (Fig. 3W). Thus, the comparable topological relationships of the developing thyroid and the precardiac mesoderm, a possible specifier of thyroid origins, between hagfish and gnathostomes implies that the direct development of the thyroid gland may represent an ancestral condition in the LCA of modern vertebrates.

In hagfish, we found that the pharyngeal region co-expressing thyroid marker genes (*Nkx2-1/2-4*, *Pax2/5/8*, and *Hhex*) was confined to the thyroid anlage in stage 51 and 53 embryos (Fig. 3B, C, E, F, H, I), which is similar to the expression patterns observed in gnathostomes [19, 20] (Additional file 1: Fig. S3F-J). Co-expression of *Nkx2-1/2-4*, *Pax2/5/8*, and *Hhex* in hagfish and gnathostomes suggests that the conserved GRN for thyroid follicle formation was present in the LCA of modern vertebrates. However, unlike gnathostomes, in which *Nkx2-1*, *Pax8*, and *Hhex* are co-expressed in the earliest thyroid anlage [19], only *Nkx2-1/2-4* was detected in the early primordia of the hagfish thyroid (stage 45; Fig. 3A’, D’, G’). Moreover, its expression domain extended rostrally towards the endodermal epithelium, up to the level of the oropharyngeal membrane, and was not confined to the prospective thyroid anlage (Fig. 3A, A’, V). In *L. camtschaticum* at Tahara’s stage 24 [27], the endostyle diverticulum was distinguishable by the broad medial expression of *Nkx2-1/2-4A* and *Nkx2-1/2-4C* genes in the pharynx (Fig. 3K, K’ and Additional file 1: Fig. S4J, J’), as previously reported [1, 2, 25]. Similar to the expression patterns in the hagfish, only the hybridized signal of *Nkx2-1/2-4* paralogues was detected in the ventral pharynx of the lamprey before the initiation of endostyle formation (Tahara’s stage 23; Fig. 3J and Additional file 1: Fig. S4I). The spatiotemporal expression patterns of *Nkx2-1/2-4* shared by hagfish and lamprey are similar to those of orthologous genes during the endostyle development in non-vertebrate chordates [13]. Furthermore, even in the hemichordate embryo which does not have an endostyle, the expression of *Nkx2-1/2-4* is evident in the pharyngeal endoderm [28]. *Nkx2-1* is focally expressed in the laryngotracheal diverticulum, in addition to the thyroid primordium; the laryngotracheal diverticulum is situated in the most caudal part of the pharynx during early development in mammals and birds [29, 30]. However, the lung and its homolog (the swim bladder) are presumed to be a synapomorphy of Osteichthyes (bony vertebrates), and thus, the expression domain of *Nkx2-1* in these respiratory organs was likely acquired after Osteichthyes and Chondrichthyes split from each other and likely does not reflect an ancestral condition of gnathostomes. Thus, our findings suggest that the broad expression of *Nkx2-1/2-4* orthologs in the ventral pharyngeal endoderm during the earliest stages of thyroid/endostyle development represents a plesiomorphic trait of chordates, which has been retained in cyclostomes, and may have been secondarily modified in crown gnathostomes where the *Nkx2-1/2-4* expression domain was not broad and regionally confined to the thyroid primordium [19].

We identified an additional *Pax2/5/8* paralogue in cyclostomes, termed *Pax2/5/8B* (Additional file 1: Fig. S4, S5). Expression signals of the lamprey *Pax2/5/8A* and *Pax2/5/8B* were not detected in the endostyle before stage 25 but were evident at stage 26 when the endostyle was almost differentiated (Fig. 3N–Q’ and Additional file 1: Fig. S4M-P). At stage 24, *Hhex* was expressed bilaterally at both ends of the diverticulum (Fig. 3S’). All thyroid marker genes were expressed in the endostyle of lamprey larvae only after stage 26 (Fig. 3M, Q, and U and Additional file 1: Fig. S4H, L, P), which were consistent with the previous studies [25, 31], but the expression of *Pax2/5/8A* and *Hhex* did not extend to the entire endostyle anlage in cross-sectional view (Fig. 3Q’, U’). The distributions of thyroid-related genes including *Pax2/5/8* and *Nkx2-1/2-4* orthologs in adult amphioxi and tunicates similarly do not extend to the entire endostyle in cross-sectional view. For example, the *Nkx2-1/2-4* ortholog is not expressed in the thyroid-equivalent elements in the ascidian *Ciona intestinalis*, and the *Pax2/5/8* ortholog is not expressed in part of the supporting elements in the cephalochordate *Branchiostoma belcheri* [12]. As co-expression of *Nkx2-1/2-4*, *Pax2/5/8*, and *Hhex* and their interactions are essential for the development of the thyroid gland in gnathostomes [19], the heterochronic, and heterogeneous expression of *Nkx2-1/2-4*, *Pax2/5/8*, and *Hhex* in the lamprey endostyle suggests that the core set of thyroid transcription factors, except for *Nkx2-1/2-4*, is not required for the endostyle development with regard to their organogenesis.

The expression of *Nkx2-1/2-4*, *Pax2/5/8*, and *Hhex* [32] orthologs in the endostyle of chordates, including the modern lampreys, may be responsible for thyroidal functions but not for its development. In the mammalian thyroid, *Nkx2-1* and *Pax8* genes are known to be involved both in organogenesis and physiological function [19, 21]. Thyroglobulin (Tg) and thyroid peroxidase (TPO) play critical roles in thyroid hormone synthesis, and the gene expression of Tg and TPO are coordinately regulated by *Nkx2-1* and *Pax8* in the thyroid [21, 33]. Likewise, TPO orthologs are co-expressed with *Nkx2-1* and *Pax8* in the endostyle of amphioxus and with *Pax8* in that of tunicates, suggesting that these regulatory genes for thyroidal function are conserved among modern chordates [12, 13]. Thus, the regionally confined expression patterns of *Nkx2-1/2-4*, *Pax2/5/8*, and *Hhex* in the endostyles of the lamprey and non-vertebrate chordates imply that these transcription factors do not orchestrate a regulatory network that forms either the endostyle or thyroid follicle. Indeed, the thyroid follicles never occur prior to metamorphosis in the modern lampreys and throughout life in non-vertebrate chordates. In other words, our findings suggest that the developmental GRNs for the endostyle and thyroid organogenesis have an independent and distinct modular nature.

The life histories of hagfish and lamprey differ conspicuously. While all modern lampreys go through a larval phase for approximately 2–8 years and reach maturity only after metamorphosis [34], modern hagfishes develop into the adult morphology directly after hatching [18]. Recently, Miyashita et al. reported that fossil stem lampreys probably lacked the filter-feeding larval phase, since fossils of yolk-sac lampreys were found with prominent eyes and a circumoral feeding apparatus [35]. The fossil evidence suggested that they may have directly developed into macrophagous predators following the yolk-dependent lecithotrophic period. Although the presence of a rudimentary endostyle in these stem lampreys cannot be ruled out, these observations cast doubt on the prevailing view that the ammocoete larva best resembles the earliest vertebrates [36, 37]. In the context of thyroid evolution, the lack of a larval phase in stem lampreys suggests an alternative hypothesis, namely, that the primitive endostyle (which is retained in modern non-vertebrate chordates) was lost in the LCA of vertebrates, and the endostyle present in modern lampreys represents secondary acquisition.

**Fig. 4:**
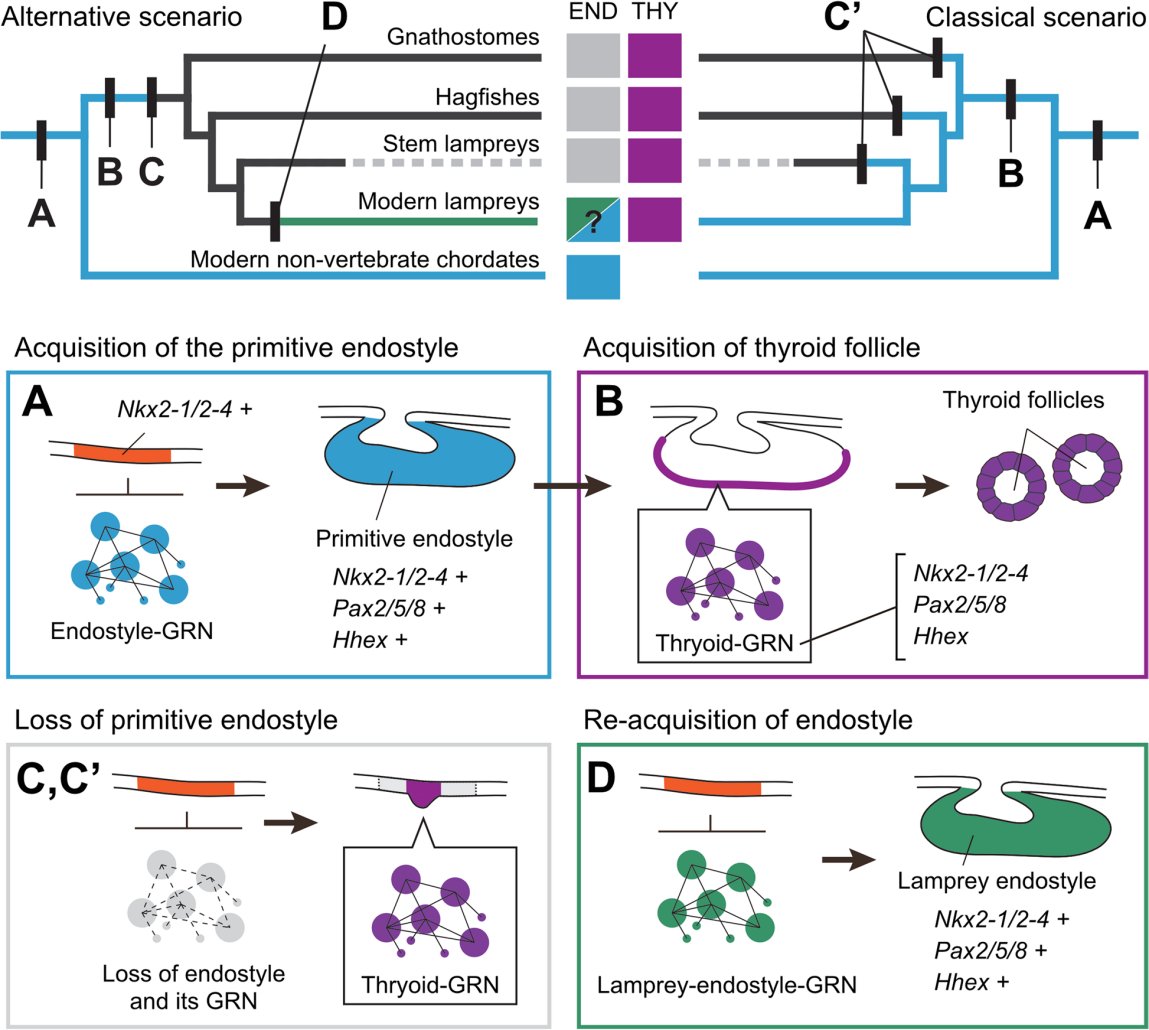
New alternative evolutionary scenario for the endostyle and thyroid gland. **A**–**D** Hypothetical independent evolutionary events that occurred during chordate evolution. Arrows indicate developmental sequence. **A** Acquisition of the primitive endostyle, which originated from the pharyngeal endoderm with broad expression of *Nkx2-1/2-4* and subordinate endostyle GRN. **B** Thyroid follicles arise from a subset of epithelial cells in the endostyle with the acquisition of thyroid GRN including *Nkx2-1/2-4*, *Pax2/5/8*, and *Hhex*. Co-existence: the primitive endostyle and thyroid follicles are not mutually exclusive in this state. **C** Anatomy and developmental module of the primitive endostyle was lost in the LCA of modern vertebrates. Crown group of cyclostomes have retained the early and broad expression of *Nkx2-1/2-4*. **C’** According to the classical hypothesis, the independent GRN of the primitive endostyle may have enabled the loss of its morphology without influencing thyroid development in each ancestor of gnathostomes, hagfishes, and possibly stem lampreys. **D** In the LCA of modern lampreys, an atavistic endostyle is secondarily acquired by recruiting a similar developmental module to form the endostyle. END, endostyle; THY, thyroid

In the present study, we found that both gnathostomes and hagfish directly developed a thyroid. There is currently no evidence that ancestral species possessed an endostyle in the branches leading to these taxa. Considering the direct development (without metamorphosis) of stem lampreys [35], our findings further raise the possibility that the endostyle was acquired secondarily in crown lampreys. Thus, we propose the following alternative evolutionary scenario for the vertebrate thyroid and endostyle (Fig. 4). The broad expression of *Nkx2-1/2-4* in the pharyngeal endoderm shared among extant cyclostomes, non-vertebrate chordates, and hemichordates represents a primitive trait of deuterostomes, and the expression domain was restricted to the ventral side of the pharynx in early chordates. A GRN involving unidentified transcription factors, instead of *Pax2/5/8* and *Hhex*, which induces the formation of the primitive endostyle, may have first occurred downstream of *Nkx2-1/2-4* (Fig. 4A). Before the acquisition of the follicular thyroid, *Pax2/5/8* and *Hhex* orthologs were probably not coupled with *Nkx2-1/2-4* and thus not involved in the endostyle or thyroid GRN, as observed in extant non-vertebrate chordates [12] (Fig. 4A). Subsequently, *Nkx2-1/2-4*, *Pax2/5/8,* and *Hhex* were organized in a colocalized expression domain, thereby establishing an interactive GRN to produce thyroid follicles in the earliest vertebrates (Fig. 4B). The acquisition of the thyroid and the subsequent loss of primitive endostyle occurred prior to the divergence of gnathostome and cyclostome lineages (Fig. 4C). An endostyle may have evolved secondarily at the base of crown lampreys in association with the filter-feeding larval stage. The cyclostome-specific (probably ancestral) broad and early expression domain of *Nkx2-1/2-4* in the pharyngeal floor (Fig. 4D) may have facilitated the co-option of an ancestral endostyle GRN.

Stem groups of vertebrates, such as *Myllokunmingia*, are considered to be microphagous suspension-feeders like modern non-vertebrate chordates [38], suggesting that the shared developmental mechanisms of the primitive endostyle were retained in these animals. Meanwhile, according to the alternative hypothesis, the fossil lamprey (*Mesomyzon mengae*) from the early Cretaceous has an ammocoete larval phase [39], suggesting that the endostyle in the modern lamprey lineage was likely acquired after the Palaeozoic.

The genetic basis of developmental mechanisms underlying endostyle formation remains largely unknown, and comparative single-cell transcriptomic analyses of the developing endostyles of non-vertebrate chordates and larval lampreys may elucidate similarities and differences in the GRN involved in morphogenesis. Furthermore, gene expression profiles in the endostyle of metamorphosing lampreys may also provide insights into the mechanisms of how the thyroid GRN is induced during the development. The novel hypothesis that the crown lamprey secondarily acquired the endostyle thus warrants further testing via future studies. According to the classical scenario, the independent nature of the GRN of the primitive endostyle from the thyroid gland may have enabled the loss of its morphology without influencing thyroid development in the respective ancestors of gnathostomes, hagfishes, and possibly some stem lampreys (Fig. 4C’). Alternatively, the LCA of modern vertebrates may have lost the primitive endostyle following the acquisition of thyroid follicles (Fig. 4C).

## Conclusions

We described the early development of the thyroid and endostyle in two extant cyclostomes and found that the histological features and the regulatory gene expression of hagfish thyroid development are nearly identical to those in gnathostomes, namely direct development. Meanwhile, different from gnathostomes, the broad pharyngeal expression of *Nkx2-1/2-4* prior to the initiation of the development of endostyle and thyroid was shared in lamprey and hagfish. Since the pharyngeal expression of *Nkx2-1/2-4* orthologs is also conserved in the living non-vertebrate chordates and hemichordates, the expression domain in the ventral pharynx is likely a synapomorphy of chordates. The endostyles and thyroid are often lumped together as a homolog, but the heterochronic and heterogeneous expression of thyroid-related genes including the *Nkx2-1/2-4* in the lamprey endostyle and nonvertebrate chordates suggests that the GRNs for the endostyle and thyroid organogenesis are likely distinct and independent as a separate developmental module. Although the plesiomorphy of lamprey endostyle remains elusive, we conclude that the spatiotemporal decoupling of these modules allowed the loss of the primitive endostyle in the vertebrate evolution. Furthermore, together with the recent fossil discovery, we question whether the lamprey endostyle is a *bona fide* plesiomorphic trait of vertebrates.

## Methods

### Sample collection and embryonic materials

*Eptatretus burgeri* is the only hagfish for which a method for obtaining fertilized eggs has been established [24]. *Eptatretus burgeri* embryos were obtained as per a previous protocol [24, 40] and fixed as described previously [17, 25, 41]. *Eptatretus atami* hatchling was sampled and fixed as described previously [41] (Additional file 1: Fig. S2H-I’). Fertilized eggs of Arctic lamprey (*L. camtschaticum*) were obtained via artificial fertilization, and the embryos were fixed with 4% paraformaldehyde. Adult cloudy catsharks (*S. torazame*) were collected from Ibaraki Prefecture, Japan. Adults and the deposited eggs were maintained in the same laboratory tank, and the embryos were fixed with 4% paraformaldehyde and Bouin’s fixative for in situ hybridization and histological observation, respectively. Embryos of cyclostomes and catshark used in this study were staged as described previously (Additional file 2: Table S1) [17, 23, 27, 42, 43].

### Histology of hagfish and catshark embryos

The tissue sections of hagfish embryos were prepared in a previous study [17]. Several peri-midline sagittal sections previously stained by in situ hybridization were used for histological observations in the present study. A 3D reconstruction of the thyroid gland and blood vessels of hagfish was added to the image initially reconstructed by Oisi et al. [17] and is presented at different angles. The 3D images of catshark embryos reconstructed by Adachi et al. [44] were recolored and are shown at different angles. Reconstructed images were obtained using Avizo software, version 9.4 (Thermo Fisher Scientific, Waltham, MA, USA).

### Molecular cloning

Previously reported partial nucleotide sequences of lamprey *Pax2/5/8A* and *Nkx2-1/2-4* orthologues of hagfish and catshark were obtained from GenBank (Accession numbers: AB079852; AB747372; AB773852) [25, 31, 45]. The sequences of hagfish *Pax2/5/8A, B,* and *Hhex*, and lamprey *Pax2/5/8B*, and *Hhex* were obtained from the previously reported transcriptome data sets from the GenBank database (SRA accession numbers: SRX2541845-SRX2541849; SRX2847491-SRX2847498) [46]. The nucleotide sequences of catshark *Pax2*, *5*, *8*, and *Hhex* were obtained from assembled transcriptome database Squalomix (https://transcriptome.riken.jp/squalomix/) [47].

The total RNA was extracted from the frozen or fresh whole embryos using the ISOGEN reagent (Nippon Gene, Toyama, Japan). The fragments of each target gene amplified with KAPA TaqExtra DNA polymerase (KAPA Biosystems, Wilmington, MA, USA) using gene-specific primer sets (Additional file 3: Table S2) were cloned into the pGEM-T-Easy vector (Promega, Madison, WI, USA) and sequenced. Digoxigenin-labeled RNA probes were synthesized by using these plasmids. The plasmids containing lamprey *Nkx2-1/2-4* paralogues (*Nkx2-1/2-4A*, *B*, *C*) generated in a previous study were used for probe synthesis [25].

### Molecular phylogenetic analysis

The molecular phylogenetic trees of vertebrate *Pax2/5/8* and *Hhex* genes were inferred via the maximum-likelihood method using PhyML3.0 (http://www.atgc-montpellier.fr/phyml/download.php) with the JTT+G4 model.

### In situ hybridization

In situ hybridization on paraffin wax-embedded sections of hagfish embryos from stages 45 to 53 (*n* = 1 biological replication) was performed as previously described [17, 25]. Whole-mount in situ hybridization of lamprey embryos from stages 23 to 26 (*n* = 8 biological replicates) and shark embryos at stage 24 (*n* = 3 biological replicates) was performed according to Kusakabe et al. [48] and Sugahara et al. [49], respectively. As negative controls, sense strand probes were used for in situ hybridization in catshark and lamprey embryos. The hybridized lamprey embryos were fixed again and embedded in a mixture of 7.5% fish gelatin (Wako Pure Chemical Industries, Osaka, Japan) and 1.5% agarose (Nippon Gene); 40-μm-thick sections were obtained using a vibratome (LinearSlicer PRO7; Dosaka EM, Kyoto, Japan).

## Supplementary Information

**Additional file 1: Fig. S1.** Endostyle and thyroid glands of chordates. **Fig. S2.** Hagfish thyroid development described by Charles Stockard and hagfish thyroid gland development during late developmental stages. **Fig. S3.** Thyroid development in catshark. **Fig. S4.** Expression patterns of cyclostome- and lamprey-specific paralogues. **Fig. S5.** Molecular phylogenetic analysis of *Pax2/5/*8 and *Hhex*.

**Additional file 2: Table S1.** Staging of cyclostome and catshark embryos used in this study.

**Additional file 3: Table S2.** Primer sets used for PCR.

## Data Availability

All data generated or analyzed during this study are included in this published article and its additional files. Newly identified cDNA sequences of catshark *Pax2*, *5*, *8*, *Hhex*, hagfish *Pax2/5/8A*, *B*, *Hhex*, and lamprey *Pax2/5/8B* have been deposited in GenBank under accession numbers LC495438 - LC495447.

## Abbreviations

LCA: Last common ancestor
GRN: Gene regulatory network
Tg: Thyroglobulin
TPO: Thyroid peroxidase
