# Supplementary material for: Thyroid and endostyle development in cyclostomes provides new insights into the evolutionary history of vertebrates
Source: BMC Biol. 2022 Apr 1;20:76. doi: 10.1186/s12915-022-01282-7 (PMC8973611; doi:10.1186/s12915-022-01282-7)
Supplement: Supplementary file 1 — Additional file 1: Fig. S1. Endostyle and thyroid glands of chordates. Fig. S2. Hagfish thyroid development described by Charles Stockard and hagfish thyroid gland development during late developmental stages. Fig. S3. Thyroid development in catshark. Fig. S4. Expression patterns of cyclostome- and lamprey-specific paralogues. Fig. S5. Molecular phylogenetic analysis of Pax2/5/8 and Hhex. [file 12915_2022_1282_MOESM1_ESM.docx]

**
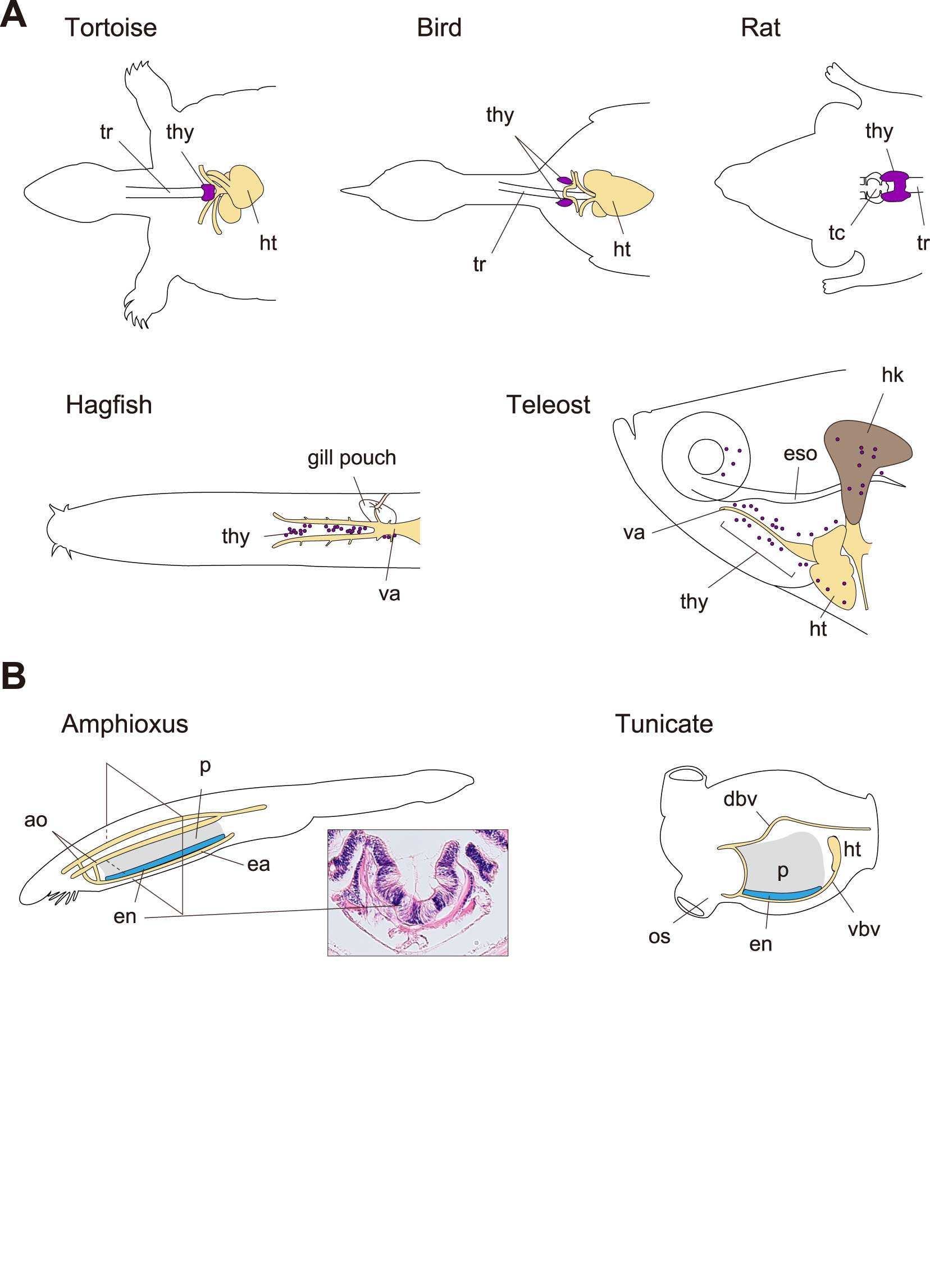
**

**Figure S1 | Endostyle and thyroid glands of chordates.** **A**, Lateral (teleost) and ventral (others) views of the vertebrate thyroid gland, colored purple. Modified drawing of *Mauremys japonica* (Tortoise), *Coturnix japonica* (Bird), *Rattus norvegicus* (Rat), *E. burgeri* (Hagfish) [4], and *Xiphophorus maculatus* (Teleost) [5]. **B**, Lateral view of the endostyle of living non-vertebrate chordates, colored in blue. Modified drawing of *Branchiostoma lanceolatum* (Amphioxus) [6] and *Corella inflata* (Tunicate) [7]. ao, aorta; ea, endostylar artery; os, oral siphon; dbv, dorsal branchial vessel; vbv, ventral branchial vessel; tr, trachea; tc, thyroid cartilage; hk, cephalic kidney; eso, esophagus. See Figs 1, 2, and 3 for other abbreviations.

**
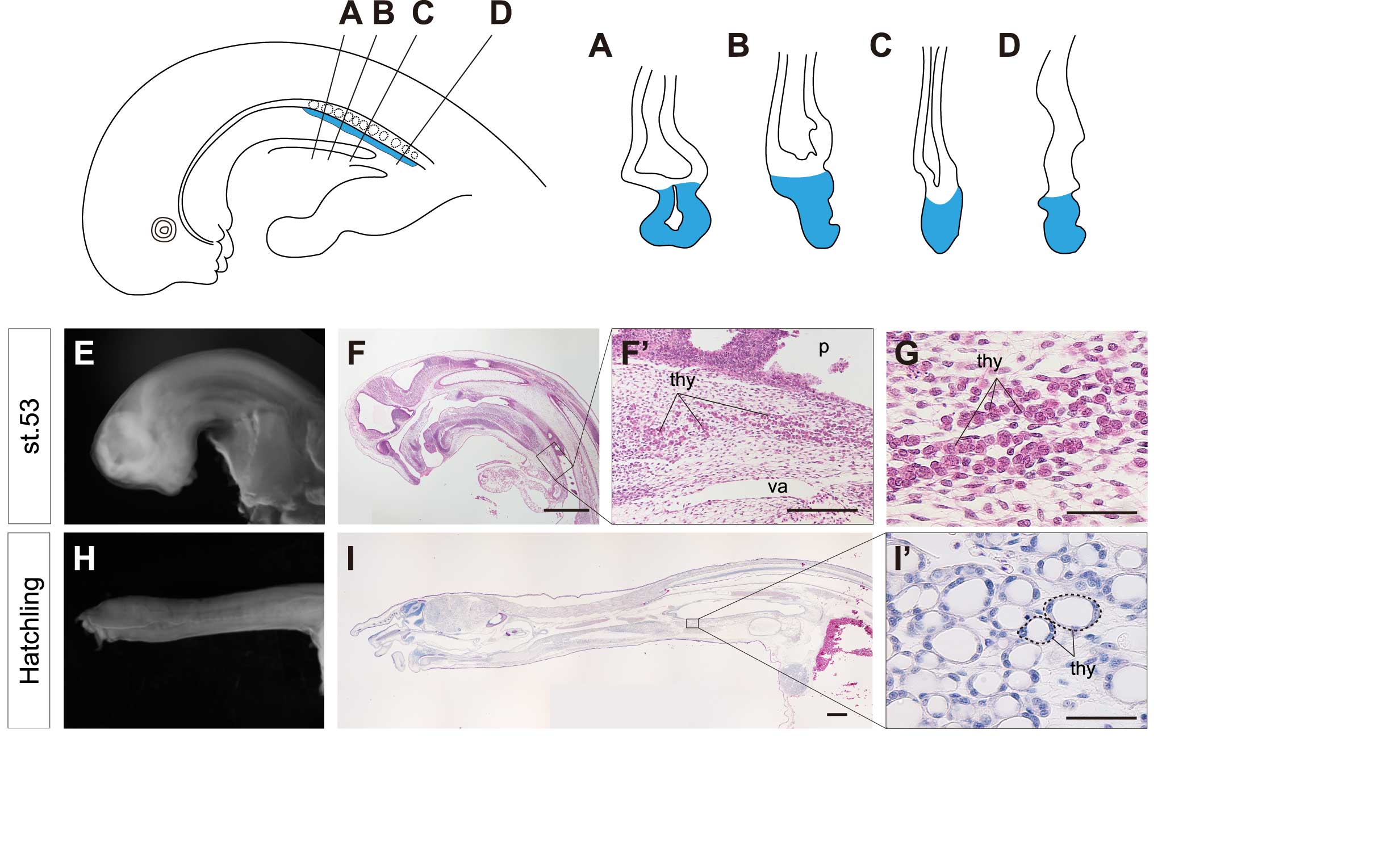
**

**Figure S2 | Hagfish thyroid development described by Charles Stockard and hagfish thyroid gland development during late developmental stages.** According to Stockard [17], the endostylar “trough” structure of *E. stoutii* thyroid developed from the pharyngeal floor throughout the entire region of gill pouches. **A-D**, transverse sections of the pharynx. Thyroid anlage is shown in blue. **E, H,** Lateral views of the fixed late pharyngula of *E. burgeri* corresponding to the developmental stage reported by Stockard and of fixed hatchlings of *E. atami* immersed in methyl benzoate before paraffin embedding. **F, F’, G,** **I, I’**, Midline sagittal sections stained with hematoxylin and eosin. **G**, Clusters of migrating thyroid cells **I’**, The area surrounded by the dashed line indicates thyroid follicles. See Figs 1 and 2 for the abbreviations. Bars, 1mm for F, I; 200 µm for F’; 50 µm for G, I’.


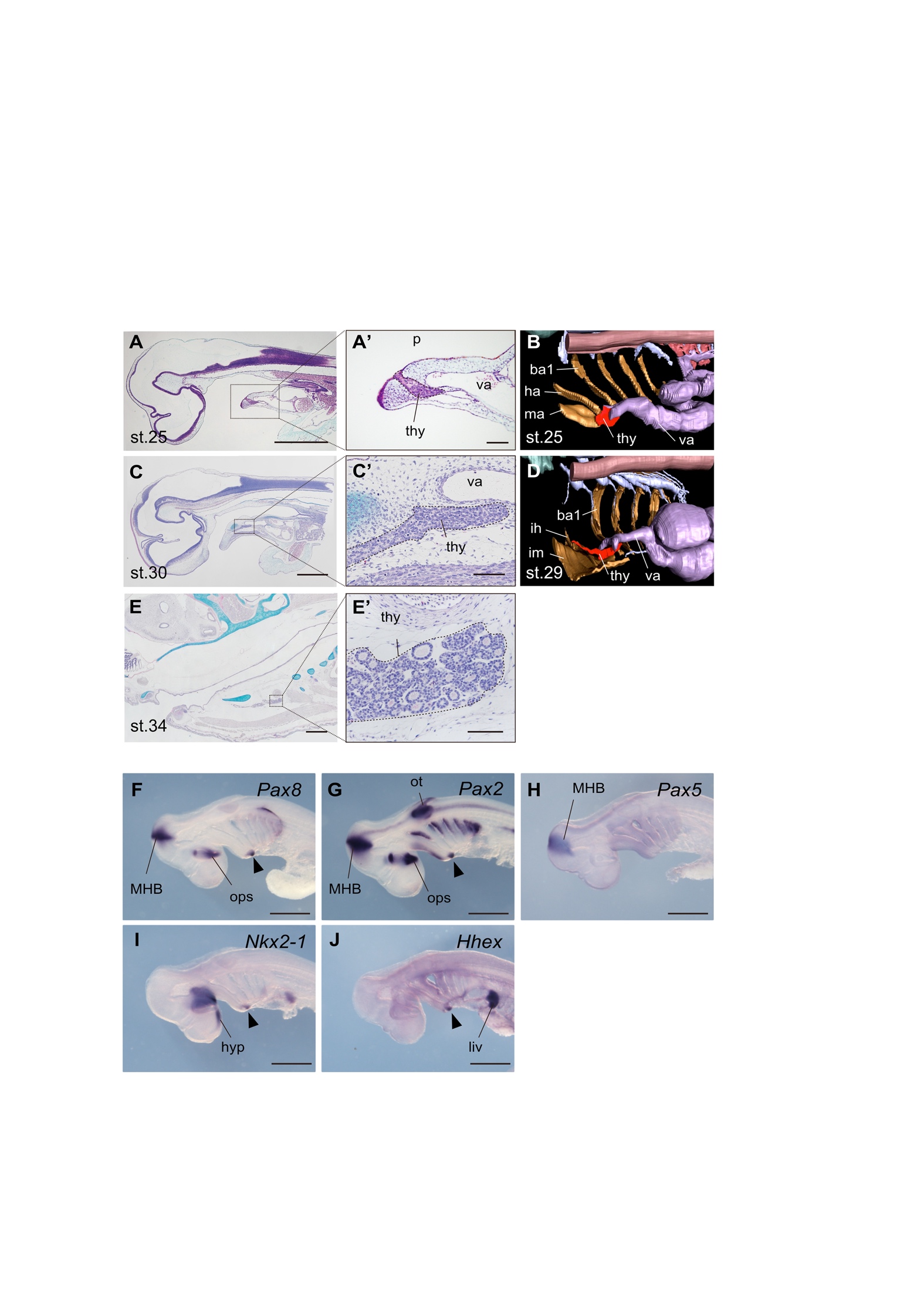


**Figure S3 | Thyroid development in catshark.** **A, A’, C, C’, E, E’,** Midline sagittal sections of an *S. torazame* embryo stained with hematoxylin, eosin, and Alcian blue. The area surrounded by a dashed line indicates the thyroid primordium. **B, D,** 3D images reconstructed from serial sections of *S. torazame* embryos using Avizo software. **F-J,** Gene expression patterns involved in early development of the thyroid gland at stage 24. Arrowheads indicate expression in the thyroid primordium. ma, mandibular arch; ha, hyoid arch; ba1, 1st branchial arch; im, intermandibularis; ih, interhyoideus; ops, optic stalk. See Figs 2 and 3 for other abbreviations. Bars, 1 mm for A, C, E, F-J; 100 µm for A’, C’, E’.

**
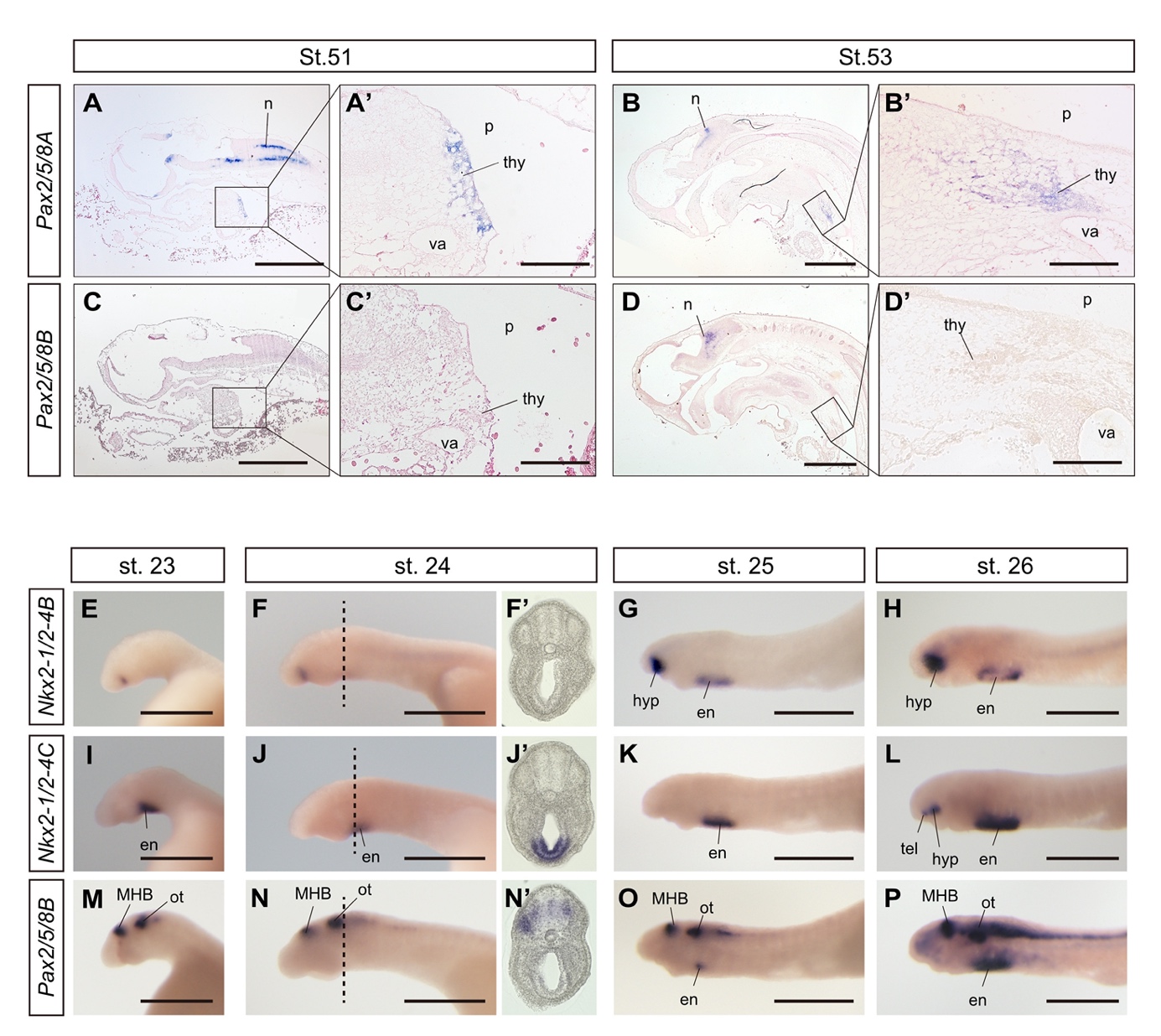
**

**Figure S4 | Expression patterns of cyclostome- and lamprey-specific paralogues. A-D’** and **E-P’**, *In situ* hybridization staining of *Nkx2-1/2-4* and *Pax2/5/8* paralogues in hagfish and lamprey embryos, respectively. n, neural tube; tel, telencephalon. See Figs 2 and 3 for other abbreviations. Bars, 1 mm (A, B, C, D), 200 µm (A’, B’, C’, D’), 500 µm (E-P)

**
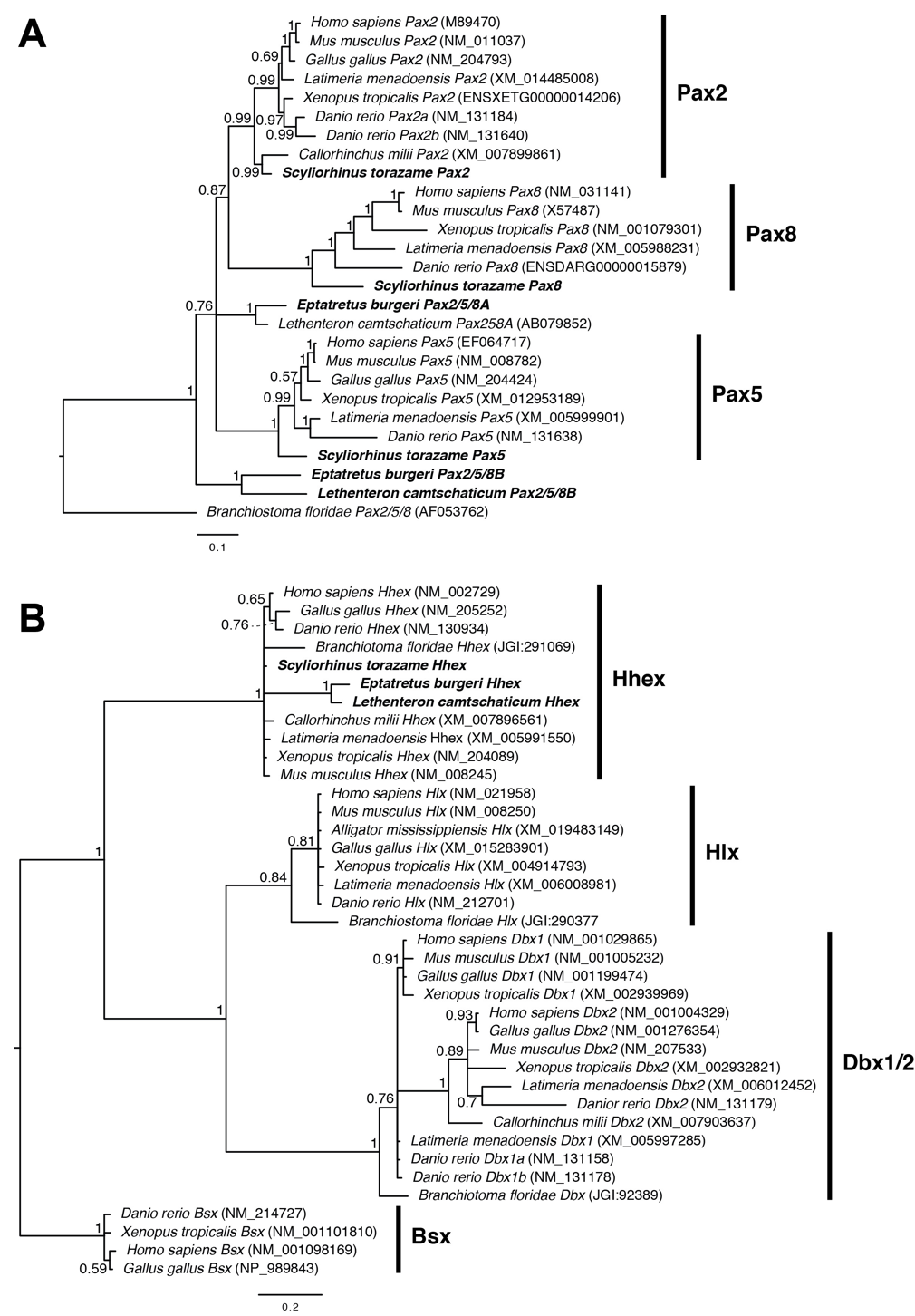
**

**Figure S5 | Molecular phylogenetic analysis of *Pax2/5/*8 and *Hhex*.** Molecular phylogenetic trees of vertebrate *Pax2/5/8* (**A**) and *Hhex* (**B**) genes including cyclostome orthologues. *Pax2/5/8A* and *Pax2/5/8B* genes are likely cyclostome lineage-specific *Pax2/5/8* paralogues. Only one *Hhex* orthologue was found in each animal. All the living gnathostomes except birds which lost *Pax8* possess *Pax2, 5 and 8*.
